# Springing of Plates

**Published:** 1854-01

**Authors:** W. S. Crane

**Affiliations:** New York


*Springing of Plates.* By Dr. W. S. Crane, New York.

In the July number of the Dental Register, I noticed an article by S. D. Muse, D. D. S., on the subject of "springing or warping of plates in soldering."

I do not pretend to have discovered any thing on this subject, which is not well understood in your city, and probably in other places; but as I have not seen a remedy for the above mentioned difficulty in print, I propose to give you a short history of my experiments on this subject, since your correspondent has expressed so much gratitude, "to all, for their kind efforts to impart useful knowledge on this subject."

I find that your correspondent depends on binding wire bent zigzag, and in binding it up in such a manner, that one would imagine that he would be greatly disappointed if his plate did not come out all right. The first time I ever saw the zigzag wire used was in Cincinnati, about seven years since, but I did not find it necessary to try it in my laboratory. I have had the pleasure of communicating to some of your western dentists, (a pleasure which is very rare, for they seldom make me a call), a much more simple method than the one your correspondent has communicated.

In the year of 1836, I commenced a new plan of inserting plates by striking chambers in them, for the purpose of producing atmospheric pressure, and it became indispensible, that the plate should be as perfect after heating as it was before, or my plan must be abandoned.

My plates were all narrow at that time, and more liable to spring in soldering, than those I make at the present time, which are about one-fourth of an inch wider. I have one of the first castings now by me, which is made of brass, and it would strike a plate jnst three-quarters of an inch wide, measuring from the lining of the front teeth to the posterior edge of the plate.

The chamber is situated on the alveolar ridge around in front, extending back to the first molars, and is about one-eighth of an inch deep, and was intended to be directly under the linings of the teeth, so that the form of the mouth should not be materially changed.

Although this plate was less than half the width of some now used, I have never had occasion to change it or make the least alteration in it whatever.

A plate covering so small a surface, must of course, be a tolerably good fit, and my efforts to prevent its warping, were indispensible, and of the utmost importance to carry out my plan. To accomplish this, I first turned my attention to the quality of the plaster. At one time the plaster was coarse, and it proved to be much better than the fine, and would stand the heat well, if slowly applied. But to make it more sure, I encircled it with wire, taking care to bury it deep, but all this precaution did not always keep the plate from warping, and like your correspondent, I turned my attention to the quality of the gold. This question was soon settled, for some would spring, and others would not, when they were all cut from the same piece.

I then concluded that I had not treated them all alike, but in what respect I could not imagine. Finally I concluded that heat was the cause of all the trouble, and I would give it enough of it, before I put it in the plaster, and warp it all I could; accordingly I gave it a soldering heat, and tried it on the cast, and found it had warped but little. I then struck it into shape, and gave it another heat of the same degree, and found it all right.

The foregoing experiments have taught me the following facts: 1st, that the heat applied in ennealing must be as great as that in soldering; 2d, that the ennealing must always be the last thing done to the plate before it is fitted to the mouth; 3d, that fine plaster will crack under the blow-pipe if it is not mixed with sand; 4th, that we want no binding wire if the heat is applied gradually; 5th, that the plate is always harder after it is swedged, than before.

[We are glad to find that Dr. Crane's views coincidc so well with our own. We refer the Dr. to Vol. 6, No. 1 of the Register. We there urge much the same reasons for the springing of plates—at least our conclusions are the same, viz., that binding wire will never prevent the springing of the plate, and the necessity of well ennealing the plate before the teeth are to be soldered. In ennealing we urge the necessity of a soldering heat, and no blow should be given to the plate after it is ennealed. We refer however to the article alluded to, for our views. In relation to the suggestion at the close of Dr. Crane's communication, we would be pleased to receive communications from any in the profession, and would always prefer that they should be of a practical character. The writer should either impress new views or show the fallacy of old ones. But we prefer they should select the subject.—Ed.]

