# Correction

**Published:** 1854-07

**Authors:** 


Dr. Taylor—*Dear Sir* :—I discover a mistake in your Journal which I regret. The article I wrote on the warping of plates you credited to another man, who was once a Dentist, but has abandoned the profession entirely. My name is John W. Crane ; you have it W. S. Crane.

[The above note should have appeared in our last, and refers to an article in the January No., Vol. VII, of the *Register.*—Ed.]

